# Prosthesis Usage and Functional Status in Upper Limb Amputees: A Prospective Cross-Sectional Study

**DOI:** 10.7759/cureus.65677

**Published:** 2024-07-29

**Authors:** Aravind P Rajan, Asem R Chanu, Srikumar Venkataraman, Upinderpal Singh

**Affiliations:** 1 Physical Medicine and Rehabilitation, All India Institute of Medical Sciences, New Delhi, IND; 2 Physical Medicine and Rehabilitation, Mahatma Gandhi Medical College and Hospital, Jaipur, IND

**Keywords:** functionality of prosthesis usage, success of prosthesis usage, prosthesis usage, opus uefs, manipal prs, upper limb amputees

## Abstract

Introduction

Amputation poses significant challenges encompassing psychological, physical, and socio-economic dimensions, impacting individuals and society at large. In India, a substantial portion of the population faces loco-motor disabilities, with amputees forming a notable segment. Prosthetic rehabilitation plays a crucial role in mitigating the consequences of limb loss, aiming to restore autonomy post-amputation.

Methods

A prospective cross-sectional observational study was conducted over 18 months, from November 2018 to May 2020, involving unilateral upper limb amputees (ULAs) aged over 18 years. A consecutive cohort of 33 patients, predominantly male (30 males and three females), with a mean age of 43 ± 12 years (median: 43 years; range: 20-67 years), was prospectively enrolled in the study. Participants had completed at least one month of post-prosthetic fitment and were actively attending outpatient or prosthetic checkout clinics. The study utilized the Manipal Prosthetic Rehabilitation Success (PRS) score and the Orthotics and Prosthetics User Survey Upper Extremity Functional Status (OPUS UEFS) score to assess prosthesis usage and functional status. Statistical analyses included descriptive statistics, Chi-square tests, Mann-Whitney U tests, Kruskal-Wallis tests, and multivariate logistic regression analysis.

Results

The study revealed insights into upper limb prosthesis usage in India, highlighting factors influencing the success and challenges faced by ULAs. Associations were found between the success of prosthesis usage and several factors: occupation type (p=0.012), the side of amputation involving non-dominant limbs (p=0.033), comfort level (p=0.002), and prosthesis weight (p=0.029). Comfort level emerged as a primary predictor of usage success. The OPUS UEFS scores indicated varying levels of satisfaction and usage patterns among participants, with some utilizing prostheses for specific tasks while others for broader activities. Comfort level demonstrated a statistically significant difference in OPUS UEFS scores, favoring comfortable prostheses (p=0.020). Additionally, the mean OPUS UEFS score for patients with satisfactory or good prosthesis use was 53 ± 11 (median: 55; range: 22-64), compared to 45 ± 13 (median: 43; range: 18 - 64) in those with poor prosthesis use, with the difference nearing statistical significance (p=0.058).

Conclusion

The study sheds light on the landscape of upper limb prosthesis usage in India, emphasizing the need for tailored interventions based on individual needs and cultural contexts. The findings underscore the importance of comfort, side of amputation involving non-dominant limbs, occupation type, and prosthesis weight in determining the success of prosthesis usage. Opportunities exist to enhance upper limb prosthetic care in India by addressing cultural nuances and refining assessment tools to better suit the Indian population.

## Introduction

Amputation inflicts a profound and multifaceted impact on patients, encompassing psychological, physical, and socio-economic dimensions. Studies have shown that amputation leads to significant emotional disturbances, such as depression and anxiety, reduced physical functionality, and substantial economic burdens on individuals and their families [1]. According to a national survey conducted in 1981, India was home to an estimated 12 million persons with disabilities, with amputees constituting 8% of the 5.4 million people facing loco-motor disabilities [2]. This underscores the significant and far-reaching consequences of limb loss on a substantial portion of the population, both in terms of individual well-being and societal implications. The goal of prosthetic rehabilitation is to mitigate the impact of limb loss, empowering patients to regain a level of autonomy comparable to their pre-amputation state. Upper limb prostheses play a crucial role in this process, serving as intricate and functional tools that enable individuals to carry out daily tasks with a degree of proficiency, distinguishing them from lower limb prostheses [3].

A significant proportion of upper limb amputee (ULA) patients exhibit reluctance or resistance to utilizing prostheses, despite the evident advantages they offer. Prosthesis usage rates for ULA range widely, spanning from 27% to 56% [4]. Notably, rejection rates of 26% for body-powered and 23% for electric prostheses were observed in adult populations, with slightly higher rates in the pediatric population at 45% and 35%, respectively [5]. In India, the fitment rates for upper limb prostheses are disappointingly low, standing at a mere 40% [6]. Interestingly, there is no significant variance in success ratings between above-elbow and below-elbow amputees, indicating a uniform challenge in achieving successful prosthetic fitment. Despite this, there is a commendable acceptance rate of around 40% for unilateral above-elbow and below-elbow amputees in adopting prosthetic solutions [7].

The landscape of upper limb prosthetics encompasses a diverse array of options, varying in functionality and cosmesis, tailored to meet users' unique needs and lifestyles. Despite a wealth of studies on upper limb prostheses conducted in Western contexts, there remains a notable dearth in the examination of the success of prosthesis usage and functional status among ULAs in emerging and developing countries, such as India. This study was undertaken to fill this critical gap and shed light on the nuanced challenges and achievements related to the adoption of upper limb prosthetics within the specific socio-cultural context of India.

## Materials and methods

This study had a prospective cross-sectional observational design within a single institutional setting. Commencing only after obtaining approval from the Institutional Ethics Committee for Postgraduate Research of All India Institute of Medical Sciences (approval number: IECPG-554/14.11.2018), the study spanned a duration of 18 months, from November 2018 to May 2020. A consecutive cohort of 33 patients was prospectively enrolled in the study. The mean age was 43 ± 12 years (median: 43 years; range: 20-67 years). Among them, there were 30 males and three females. Additionally, four patients had a history of using more than one prosthesis. The study included unilateral ULAs aged >18 years who had completed at least one month post-prosthetic fitment and were actively attending outpatient and/or prosthetic checkout clinics. Other inclusion criteria were non-congenital amputation and the ability to comprehend English. The exclusion criteria were impaired cognitive function or intellectual disability, unwillingness to participate and give informed written consent, and blind individuals.

Data collection

Clinical history, baseline demographic data, and a general physical examination of all the subjects were recorded, following which a detailed residual limb examination was performed. To evaluate prosthesis usage and functional status, the study employed the Manipal Prosthetic Rehabilitation Success (PRS) score [7] and the Orthotics and Prosthetics User Survey Upper Extremity Functional Status (OPUS UEFS) score [8], respectively. The analysis assessed the relationship between patient demographics, amputation, and prosthesis-related characteristics with both Manipal PRS and OPUS UEFS scores. The success of prosthesis usage was defined as a Manipal PRS score of ≥3. The OPUS UEFS scores evaluated patients' ability to perform 23 specific activities, graded on a four-point scale: 0 (unable to perform), 1 (difficult), 2 (easy), and 3 (very easy). Scores ranged from 0 to 69.

Statistical analysis

Various descriptive statistics, such as mean ± standard deviation (SD), median (range), proportions, and percentages, were used to describe the patient demographics, amputation-related characteristics, and prosthesis characteristics. Categorical variables were analyzed using the Chi-square or Fisher’s exact test, as applicable, while quantitative variables between the two groups were compared with the Mann-Whitney U test. The Kruskal-Wallis test, with Bonferroni correction, was utilized for comparisons across multiple groups for quantitative variables. Multivariate logistic regression analysis was performed to identify the independent predictors for the success of prosthesis usage. A two-tailed p-value of <0.05 was considered statistically significant for all analyses. SPSS Statistics version 26 (IBM Corp. Released 2019. IBM SPSS Statistics for Windows, Version 26.0. Armonk, NY: IBM Corp.) software was utilized for statistical computations.

## Results

Manipal prosthetic rehabilitation success score

Nineteen ULAs demonstrated good or satisfactory prosthesis usage, indicated by a Manipal PRS score of ≥3, while 14 exhibited poor prosthesis usage with a Manipal PRS score of <3. Among the various patient demographics, occupation emerged as significantly associated with the success of prosthesis usage (p=0.012) (Table 1). Regarding amputation-related characteristics, the side of amputation involving the non-dominant limb showed a significant association with the success of prosthesis usage (p=0.033) (Table 2). Additionally, among prosthesis characteristics, both comfort level on prosthesis use and subjective weight of the prosthesis were significantly associated with the success of prosthesis usage (p=0.002 and p=0.029, respectively) (Table 3). On multivariate analysis, comfort level with prosthesis use was found to be the independent predictor of success with prosthesis usage (p=0.049) (Table 4).

**Table 1:** Manipal PRS and OPUS UEFS stratified by patient demographics * Bonferroni corrected significance level between “not earning” and “>50,000 INR.” Other pairwise comparisons were not significant (p≥0.250). # Bonferroni corrected significance level between “physical laborer” and “desk job.” Other pairwise comparisons had a p-value of ≥0.106. Manipal PRS: Manipal Prosthetic Rehabilitation Success, OPUS UEFS: Orthotics and Prosthetics User Survey Upper Extremity Functional Status

Characteristic	N	Manipal PRS		OPUS UEFS scores	
		Satisfactory or good (N)	p-value	Mean ± SD (median, range)	p-value
Gender					
Male	30	18	0.561	50 ± 13 (55, 18-64)	0.113
Female	3	1		40 ± 9 (41, 31-48)	
Age					
20-40 years	15	7	0.338	48 ± 9 (50, 34-60)	0.384
40-60 years	15	9		51 ± 16 (57, 18-64)	
>60 years	3	3		48 ± 8 (44, 43-58)	
Income (INR)					
<10,000	8	5	0.125	48 ± 11 (48, 34-64)	0.014 (0.014)*
10,000-20,000	3	1		49 ± 7 (50, 42-55)	
20,000-50,000	3	1		59 ± 5 (60, 54-64)	
>50,000	6	6		61 ± 5 (64, 55-64)	
Not earning	13	6		42 ± 14 (43, 18-60)	
Education					
No formal education	2	2	0.464	51 ± 9 (51, 45-57)	0.098
School level	17	8		44 ± 14 (43, 18-64)	
Graduate level	14	9		55 ± 9 (56, 40-64)	
Occupation					
Physical labourer	5	4	0.012	37 ± 17 (43, 18-57)	0.015 (0.086)^ #^
Business	4	0		40 ± 8 (39, 34-50)	
Desk job	13	10		59 ± 6 (60, 42-64)	
Homemaker	1	1		-	
Student	5	1		49 ± 9 (48, 40-59)	
Jobless	3	1		50 ± 14 (55, 34-60)	
Others	2	2		47 ± 4 (47, 44-50)	
Marital status					
Married	23	15	0.257	49 ± 14 (50, 18-64)	0.893
Unmarried	10	4		50 ± 9 (55, 34-60)	
Limb dominance					
Right	25	13	0.416	45 ± 12 (45, 18-60)	<0.001
Left	8	6		62 ± 3 (64, 55-64)	

**Table 2:** Manipal PRS and OPUS UEFS scores stratified by amputation-related characteristics ROM: range of motion, Manipal PRS: Manipal Prosthetic Rehabilitation Success, OPUS UEFS: Orthotics and Prosthetics User Survey Upper Extremity Functional Status

Characteristic	N	Manipal PRS		OPUS UEFS scores	
		Satisfactory or good (N)	p-value	Mean ± SD (median, range)	p-value
Side of amputation					
Dominant limb	21	9	0.033	47 ± 14 (48, 18-64)	0.213
Non-dominant limb	12	10		53 ± 10 (56, 31-64)	
Cause of amputation					
Trauma	25	13	0.597	49 ± 14 (55, 18-64)	0.700
Cancer	3	2		54 ± 7 (55, 43-61)	
Others	5	4		46 ± 4 (45, 43-50)	
Duration of amputation					
≤1 year	5	4	0.366	54 ± 3 (55, 50-57)	0.566
>1 year	28	15		48 ± 13 (49, 18-64)	
Level of amputation					
Forequarter	1	1	0.574	-	0.547
Shoulder disarticulation	2	2		49 ± 7 (49, 44-54)	
Trans-humeral	12	7		54 ± 9 (57, 40-64)	
Trans-radial	16	7		45 ± 15 (47, 18-64)	
Wrist disarticulation	1	1		-	
Partial hand	1	1		-	
Residual limb length (% of sound limb length)					
≤50%	23	13	1.000	49 ± 14 (50, 18-64)	0.923
>50%	10	6		50 ± 10 (55, 34-61)	
Residual limb ROM					
Normal	22	13	0.681	52 ± 11 (55, 31-64)	0.139
Restricted	10	6		44 ± 15 (46, 18-61)	
Increased	1	0		-	
Scar status					
Adherent and painless	2	2	0.507	44 ± 18 (44, 31-57)	0.499
Adherent and painful	2	1		49 ± 8 (49, 43-54)	
Non-adherent and painless	25	15		50 ± 13 (55, 18-64)	
Non-adherent and painful	4	1		45 ± 9 (45, 34-45)	

**Table 3:** Manipal PRS and OPUS UEFS scores stratified by prosthesis characteristics Manipal PRS: Manipal Prosthetic Rehabilitation Success, OPUS UEFS: Orthotics and Prosthetics User Survey Upper Extremity Functional Status

Characteristic	N	Manipal PRS		OPUS UEFS scores	
		Satisfactory or good (N)	p-value	Mean ± SD (median, range)	p-value
Type of prosthesis					
Cosmetic	17	10	0.546	49 ± 13 (54, 18-64)	0.966
Body-powered	14	7		49 ± 13 (53, 22-64)	
Myoelectric	2	2		50 ± 8 (50, 44-55)	
Time from amputation to prosthesis fitment					
≤1 year	21	12	1.000	53 ± 11 (55, 31-64)	0.083
>1 year	12	7		44 ± 14 (44, 18-61)	
Time since prosthesis fitment					
≤1 year	7	6	0.195	54 ± 5 (55, 45-61)	0.416
>1 year	26	13		48 ± 14 (49, 18-64)	
Number of prostheses used					
1	23	12	0.455	51 ± 10 (55, 34-64)	0.357
>1	10	7		45 ± 17 (47, 18-64)	
Fitment center					
Current institute	14	8	1.000	52 ± 13 (56, 31-64)	0.257
Elsewhere	19	11		47 ± 12 (50, 18-61)	
Pain on prosthesis use					
Present	6	1	0.062	48 ± 13 (48, 34-64)	0.707
Absent	27	18		50 ± 13 (55, 18-64)	
Comfort level on prosthesis use					
Comfortable	18	15	0.002	53 ± 13 (56, 18-64)	0.020
Uncomfortable	15	4		45 ± 10 (43, 31-64)	
Subjective weight of prosthesis					
Heavy	13	4	0.029	49 ± 10 (50, 34-64)	0.505
Light	20	15		50 ± 14 (55, 18-64)	

**Table 4:** Multivariate analysis for prediction of success of prosthesis use CI: confidence interval

Characteristic	Odd’s ratio (95% CI)	p-value
Occupation	1.050 (0.567-1.945)	0.876
Side of amputation	4.623 (0.564-37.865)	0.154
Comfort level on prosthesis use	0.123 (0.015-0.993)	0.049
Subjective weight of prosthesis use	2.826 (0.424-18.825)	0.283

Orthotics and prosthetics user survey upper extremity functional status score

In our study sample, the mean OPUS UEFS score was 49 ± 13 (median: 54; range: 18 - 64). Notably, OPUS UEFS scores exhibited significant variations across different income groups (p=0.014), occupations (p=0.015), and limb dominances (p<0.001) among various patient demographics. Through pairwise comparison with Bonferroni correction, we found that the difference in OPUS UEFS scores between patients earning >50,000 INR and those "not earning" remained statistically significant (p=0.014) (Table 1). However, OPUS UEFS scores across patients with different amputation-related characteristics did not reveal any significant statistical differences (p≥0.139) (Table 2). Regarding prosthesis characteristics, the comfort level during prosthesis use demonstrated a statistically significant difference in OPUS UEFS scores, favoring comfortable prostheses (p=0.020).

Furthermore, the mean OPUS UEFS score in patients who had satisfactory or good prosthesis use (Manipal PRS score ≥3) was 53 ± 11 (median: 55; range: 22-64), in contrast to 45 ± 13 (median: 43; range: 18-64) in those with poor prosthesis use (Manipal PRS score <3). The difference showed a trend toward statistical significance (p=0.058).

## Discussion

The study predominantly comprised male participants (30/33; 90%), echoing findings from the Indian epidemiological survey of amputees [2]. The majority fell within the 20-60 year age bracket (30/33; 90%), likely due to the heightened exposure to hazardous work environments within this age group [9]. Corresponding with prior Indian studies by Agarwal and Goel [10] and Singh and Verma [6], the primary cause of amputations in our sample was trauma (25/33; 76%). Notably, a significant proportion were either trans-humeral or trans-radial amputees (28/33; 84%), contrasting with the predominance of finger amputations observed in the Indian population-based study by Singh and Verma [6]. This variance could stem from the fact that participants in our study sought prosthetic fitting or repair at our center, possibly indicating more substantial functional loss and disfigurement compared to finger amputees in their study.

In our study, the type of occupation demonstrated a significant association with the success of prosthesis usage. Specifically, 10 out of 13 patients with desk jobs, four out of five physical laborers, two out of two patients with the "others" occupation category, and one homemaker out of one showed good or satisfactory Manipal PRS scores. Conversely, none of the patients in the business occupation, one out of five in the student category, and one out of three in the jobless category achieved good or satisfactory Manipal PRS scores. The observed association between occupation type and the success of prosthesis usage likely stems from various factors related to the demands and nature of different occupations, as well as the individual's adaptability and requirements. Factors such as the level of physical activity, manual dexterity required, and adaptability to prosthesis use within the work environment might contribute to the observed associations. Furthermore, we found that the side of amputation involving the non-dominant limb was associated with the success of prosthesis usage. Specifically, 10 out of 12 patients in this group exhibited good or satisfactory Manipal PRS scores, whereas only nine out of 21 ULAs involving the dominant limb achieved the same. This finding may be attributed to various factors. The dominant limb, accustomed to precise movements, may pose challenges in adapting to prosthetic use, whereas the non-dominant limb, less relied upon for intricate tasks, may offer greater flexibility for adaptation. Additionally, the demands placed on each limb and psychological factors, such as confidence and motivation, likely contribute to the observed association, with individuals feeling more comfortable and confident in using a prosthesis on their non-dominant limb. The findings of the current study align broadly with those of Salminger et al. [11], who found that amputations involving the non-dominant limb had lower prosthesis rejection rates. However, these results contrast with the study by Østlie et al. [12], who could not establish an association between the side of amputation and prosthesis rejection rates. The discrepancy may be attributed to differences in study populations, methodologies, and the criteria used for defining and measuring prosthesis success and rejection.

The comfort level of prosthesis use emerged as another crucial factor influencing the success of prosthesis usage, as expected. Notably, 15 out of 18 patients using comfortable prostheses demonstrated good or satisfactory Manipal PRS scores, contrasting with only four out of 15 for those using uncomfortable prostheses. Moreover, the subjective weight of the prosthesis was also associated with the success of prosthesis usage. Among ULA patients, 15 out of 20 using lightweight prostheses achieved good or satisfactory Manipal PRS scores, compared to four out of 13 in the heavy-weight prosthesis category. This finding is intuitive, considering the impact of prosthesis weight on comfort and usability. Our findings resonate with those of Salminger et al. [11], Schultz et al. [13], and the systematic review by Smail et al. [14], who emphasized the critical importance of comfort level and prosthesis weight in the successful use of prostheses among ULAs. These studies underline that comfort and the manageable weight of prostheses are key determinants of user satisfaction and continued use, reinforcing the notion that ergonomic design and user-centered customization are pivotal to prosthetic success.

It's intriguing to discover that in our study, comfort level during prosthesis use emerged as the sole independent predictor for the success of prosthesis usage, diverging from findings by Raichle et al., which linked prosthesis use in ULAs to factors like level of amputation, phantom limb pain, and marital status [4]. Notably, while Raichle et al. assessed prosthesis use in terms of hours per day or days per month, our study utilized a more objective and standardized measure, the Manipal PRS score. Moreover, our study conducted multivariate analysis to mitigate confounding variables and identify independent predictors of prosthesis usage success. Unlike Raichle et al.'s findings [4], our study did not establish any association between the level of amputation and the success of prosthesis usage, aligning with Bhaskaranand et al.'s observations, which similarly found no significant differences in Manipal PRS scores across various levels of amputation in ULAs [7].

The study sample exhibited an average OPUS UEFS score of 49 ± 13 (median: 54; range: 18-64), indicating generally favorable functionality as perceived by the prosthetic users. In our study, ULAs with higher incomes (>50,000 INR) demonstrated higher OPUS UEFS scores, likely attributed to the combined factors of superior prosthetic care access, increased engagement in rehabilitation and activities, reduced stressors, and financial resources. Our findings resonate with the existing literature, which identifies lower income as a significant barrier to accessing optimal healthcare and quality prosthetic rehabilitative care. Studies by Nagaraja et al. [15] and Brack et al. [16] have highlighted how financial constraints limit access to advanced prosthetic devices and comprehensive rehabilitation services, thereby affecting overall outcomes and quality of life for ULAs. This underscores the need for policies aimed at reducing financial disparities to improve prosthetic success rates among lower-income populations. Additionally, left limb dominant patients exhibited higher OPUS UEFS scores, regardless of amputation side, reflecting a possible blend of inherent neurological advantages, adaptive techniques, and heightened motor skills enhancing prosthetic functionality. Furthermore, ULAs reporting comfortable prostheses displayed higher OPUS UEFS scores, possibly due to enhanced consistency in use and participation in activities, alongside the possibility of improved proprioception and satisfaction.

Among the 14 bimanual activities assessed in the OPUS UEFS, seven could be performed with one hand, while the remaining set of items posed greater difficulty or were impossible to execute single-handedly [17]. As anticipated, a higher proportion of respondents found the easier set of bimanual and mono-manual activities "very easy," while fewer found the tougher bimanual tasks easy to perform. However, two exceptions were noted. Firstly, despite Q22 (stir a bowl) being classified as a bimanual activity typically challenging for amputees, the majority (28/33, 84.8%) reported it as "very easy." This discrepancy could stem from cultural differences in interpreting the task; while Western authors may envision stirring dough in a large bowl, Indians may perceive it as stirring liquid in a small bowl, a simpler mono-manual task. Secondly, Q16 (use a key in a lock), considered an easy mono-manual activity by Burger et al. [17], elicited relatively fewer respondents in our study, reporting it as "very easy" (11/33, 33.3%) or "easy" (7/33, 21.2%). This disparity could arise from differing cultural contexts; whereas Western authors might envision a door lock, Indians commonly use padlocks to secure main doors, which often require stabilization with one hand before inserting the key with the other.

It is crucial to recognize that the OPUS UEFS aims to assess a patient's upper limb functional status rather than solely quantifying the prosthesis' functioning. The questionnaire also accounts for whether tasks were performed with or without prosthetic assistance. For instance, Q17 (carry a laundry basket) saw the highest number of ULAs claiming prosthesis use, yet only 36.4% (12/33) utilized one, often opting for body-powered prostheses primarily for cosmetic purposes. Notably, a 64-year-old trans-humeral amputee with a non-dominant limb amputation used a myoelectric prosthesis for two years solely for cosmetic reasons, highlighting a disconnect between expectations and outcomes. Similarly, a 59-year-old lady with a non-dominant trans-radial amputation found satisfaction with her LN4 prosthetic hand [18] over 12 years, though she primarily used it for only two tasks in the OPUS UEFS assessment (cutting meat with a knife and fork and carrying a laundry basket). Upon further inquiry, she revealed unconventional uses, such as tying a knife to the prosthesis to cut grass for her cattle or using it for support while carrying firewood, underscoring both a lack of awareness regarding appropriate prosthesis uses and the limitations of the OPUS UEFS in rural Indian settings, where tasks like cutting grass or carrying firewood hold greater relevance than traditional assessment criteria such as tying shoelaces or attaching end zippers in a jacket.

While our study contributes valuable insights, it's important to acknowledge its limitations, with the small sample size being the most prominent. Moreover, the absence of specific upper limb functionality measures tailored to the Indian population necessitated the use of the OPUS UEFS scale, albeit with reservations regarding its ability to fully capture functionality in Indian settings, especially rural contexts. Additionally, to minimize potential recall bias, assessments for participants using multiple prostheses focused solely on their current prostheses, ensuring a more accurate evaluation of their usage and functionality.

## Conclusions

Our study illuminates the landscape of upper limb prosthesis usage in India, uncovering pivotal factors influencing both success and hurdles. The associations between occupation type, side of amputation involving non-dominant limbs, comfort level, and prosthesis weight with usage success offer valuable insights for tailored interventions. Notably, comfort level emerged as the primary predictor, underscoring its pivotal role in facilitating successful prosthetic adoption. Despite the promising functionality perceptions reflected in OPUS UEFS scores, cultural nuances in task interpretation were evident, warranting context-specific approaches. While acknowledging limitations like a modest sample size and reliance on Western-centric tools, our findings signal opportunities for refining upper limb prosthetic care within the Indian context.
